# Editorial: Kampo Medicine in a Modern Context: Ethnopharmacological Perspectives

**DOI:** 10.3389/fphar.2022.971254

**Published:** 2022-09-02

**Authors:** Kenny Kuchta, Silke Cameron

**Affiliations:** ^1^ Forschungsstelle für Fernöstliche Medizin, Department of Vegetation Analysis and Phytodiversity, Albrecht von Haller Institute of Plant Sciences, Georg August University, Göttingen, Germany; ^2^ Clinic for Gastroenterology and GI-Oncology, University Medical Center Göttingen, Georg August University, Göttingen, Germany; ^3^ Department for Gastroenterology and General Internal Medicine, Clinic Hann. Münden, Hann. Münden, Germany

**Keywords:** Kampo (traditional Japanese herbal medicine), Ethnopharmacology, cultural evolution, dementia—Alzheimer disease, supportive cancer therapy, personalised therapy, COVID-19 therapeutics, ICD-11 (international classification of diseases)

The word “Kampo” (漢方) is used to denote the traditional academic phytotherapy of Japan. This term, which can be translated as “Method of Han-Dynasty-China”, was coined in the 19th century to distinguish the long established traditional medicine from the new influx of Western medicine.

In contrast to the pre-existing system of purely orally transmitted “Minkanyaku” (民間薬) practiced by non experts, Kampo medicine was established through the 6th to the 10th century AD as a specialised academic profession. This was possible through the import of books - such as the Shoukanron (傷寒論/Chin. Shanghan Lun) and the Shinnouhonzoukyou (神農本草経/Chin. Shennong Bencaojing)—and immigration of trained experts from China and Korea. In the following centuries–especially from the 17th to the 19th century during the seclusion of Japan, Japanese traditional medicine evolved largely independent from external influences. Thus, Ancient Chinese Medicine (ACM) was at the origin of a divergent cultural evolution leading to several distinct contemporary forms of Eastern Medicine such as Japanese Kampo, Traditional Chinese Medicine (TCM) and Korean Medicine (KM) [Kuchta K. Zeitschrift für Phytotherapie 2014; 35 (02): 79–84].

In biological evolution, the process of divergent evolution of one species into several daughter species as an adaptation to distinct environments is well known. The general preconditions of evolution 1) Replication, 2) Mutation, and 3) Selection of information [Darwin C (1871). The Descent of Man and Selection in Relation to Sex. Photoreproduction of the 1871 Edition (Princeton, New Jersey London: Princeton University PressJ. Murray), 60–61] drive not only the evolutionary development of genetic information, but also of cultural systems: They are omnipresent in all aspects of human culture, where knowledge (information) can easily be replicated (learning), mutated (innovation), and is subject to selection. The resulting body of accumulated knowledge and engrained behaviours is referred to as “tradition” or “culture” and the process of its development and distribution can be referred to as “cultural evolution”. In the case of traditional medicines and herbal remedies, the evolutionary pressure that drives their cultural evolution is the survival benefit of effective treatments. As any successful therapy depends on 1) human physiology and 2) the pathophysiology of the disease, these two factors form the equivalent of Darwinian evolutionary pressure in the cultural evolution of traditional medicine. Thus - although traditional medicine is not based on the modern knowledge of human physiology, biochemistry or genetics - the underlying information for safety and efficacy is “imprinted” onto traditional theories and practice by the cultural evolution process itself. Traditional medicines therefore exhibit an *a priori* internal structure that already corresponded to human pharmacology and physiology, long before these were scientifically understood. Whilst the “Bench to Bedside” and the “Bedside to Bench” approaches have been established also for medicinal (plant) research, we propose a third, novel approach: “From Tradition to Pathogenesis”. Several examples are given where the understanding of empirical treatments leads to the understanding of the pathogenesis of disease. This understanding of cultural evolution in the context of traditional medicine and its application to modern pathophysiology might help to clarify as of yet unknown pathomechanisms of disease, or at least formulate a hypothesis which can be examined at a later stage of research [Kuchta and Cameron].

As Kampo has developed into its own distinct form of Eastern Medicine, Kampo diagnostics and anamnesis developed [Kuchta and Cameron]. E.g. in Kampo medicine, there exists an important system of abdominal diagnosis called Fukushin (腹診). By applying pressure to the abdomen of the patient, the physician can read the patients’ physical state from the “patterns” of firmness or softness and thus choose a suitable Kampo formulation. Traditional Kampo anamnesis aims to correlate symptoms and abdominal “patterns” to the respective prescription formulas. In the present volume, Yakubo et al. report on the development of a Fukushin simulator, a teaching tool that reproduces the important abdominal “patterns” that doctors encounter in clinical practice. The various Fukushin models simplify the teaching of these inherently “hands on” concepts. In recent years, Kampo Fukushin “pattern” diagnosis and the corresponding anamnesis have found their way into the new ICD-11 framework of the WHO alongside several related concepts from TCM [Wu X. et al.].

With the establishment of the current form of the Japanese national health insurance system in the late 1960s the status of Kampo as standardized academic medicine was legally established. Currently, 148 Kampo prescriptions are covered by the national insurance system. The health insurance thus covers these Kampo medicines when prescribed by any medical doctors - not only by dedicated Kampo therapists. Further, official approval standards for over-the-counter Kampo products have been established for 294 formulations [Kuchta and Cameron].

As such, Kampo constitutes an integral part even of the most innovative forms of therapy such as the Japanese response to the current COVID-19 pandemic. In the present volume, Ogawa-Ochiai et al. present data from a retrospective observational study documenting the immunological and preventive effects of the Kampo prescriptions Hochuekkito (補中益気湯) and Kakkonto (葛根湯) against COVID-19 in healthcare workers.

Takayama et al. present a review of all currently available data from case reports and ongoing clinical trials on the prevention and treatment of COVID-19 patients with Kampo medicine as a whole. Building on the successful use of Kampo during the Spanish flu one century earlier, the authors were able to document numerous successful Kampo therapies for the current pandemic.

Moving from clinical research to laboratory research, Kakimoto et al. used an *in vitro* model system to demonstrate that several well established Kampo formulas that are commonly used in the treatment of respiratory symptoms of the common cold were able to successfully suppress SARS-CoV-2 infection. Here, Maoto (麻黄湯) was the most effective among the tested Kampo formulas, and Ephedrae herba according to the Japanese Pharmacopoeia (JPh XVIII) was the most effective among the tested individual herbal drugs. Ephedrae herba is also one of the main component drugs of Kakkonto (葛根湯/see above), a prescription that was demonstrated by Saito et al. to inhibit rhinovirus induced cytokine production in primary cultures of human nasal epithelial cells.

Whilst Kampo medicines enhance the immune systems’ self-defence and might thus be used to treat acute infections, their main strength lies in the treatment (and prevention) of chronic disease, where microcirculation, the immune system and healing properties play a role.

In an ageing society an increased incidence of cancer constitutes a major challenge to the public health system. Even though Kampo medicine cannot replace chemotherapy, it has proven its invaluable potential as a supportive therapy.

Chemotherapy-induced oral ulcerative mucositis for instance is one of the most common and challenging side effects of anti-tumour chemotherapy. As Miyano et al. have demonstrated, the Japanese Kampo medicine Hangeshashinto (半夏瀉心湯) improves this condition markedly. In this volume, they provide evidence suggesting that Hangeshashinto enhances human oral keratinocyte migration by up-regulating the chemokine protein stromal cell-derived factor 1, also known as C-X-C motif chemokine 12 (CXCL12), via extracellular signal-regulated kinase.

Furthermore, Kampo treatments for dementia - one typical case of a long term chronic disease - deserve special mention. In their review, Kuchta et al. demonstrate the efficacy of the traditional prescription Kamiuntanto (加味温胆湯) as a useful treatment option. One of its component drugs Polygala radix (JPh XVIII) appears to be the biggest individual contributor to this activity. As similar effects could also be demonstrated for Saffron - the dried red stigmata of the flowers of *Crocus sativus* L. - the combination of the two active principles as Kamiuntantokabankoka (加味温胆湯加番紅花) proved most promising as treatment option for dementia. As an alternative approach for the treatment of dementia Wenger et al. have reviewed Kampo prescriptions that traditionally contain cannabis (*Cannabis sativa* L.) as its major neurological active cannabinoids cannabidiol (CBD) and tetrahydrocannabinol (THC) have long been identified as important anti-dementia drug candidates. Consequently, Shakanzoto (炙甘草湯) the “long-term use [of which] is believed to promote youthfulness and lucidness” and which is further used against typical afflictions of the elderly such as “shortness of breath and palpitations with constipation-like symptoms, lack of nutrients, dry skin, and fatigue” seems to be one of the most promising candidates for future wide spread use in dementia therapy. It should be emphasised that Kampo prescriptions based on *Cannabis sativa* L. generally carry a far smaller risk of addiction than similar Western medicinal products as in Kampo only the relatively less addictive seeds are used.

Overall, undesired drug effects in Kampo are extremely rare and new methods to prevent and counteract such effects are constantly being developed. Liquorice (JPh XVIII) is one of the most common crude drugs in traditional Japanese Kampo medicine and famed for numerous anti-inflammatory and anti-infective properties. However, in high dosages its major constituent glycyrrhizin can cause pseudohyperaldosteronism as a side effect. Ishiuchi et al. have identified 18β-glycyrrhetyl-3-O-sulfate as a glycyrrhizin metabolite in a rat model and present convicting evidence that this compound, which is mainly detected in serum of pseudohyperaldosteronism patients, is the most promising causative agent of this adverse effect. They have successfully established an ELISA system able to monitor its blood concentration in patients taking liquorice. This new technology will further strengthen the predisposition of Kampo as a highly personalised safe and effective therapy.

In summary, Kampo medicine is well-established in conjunction with Western medicine in Japan, especially for the inflammation and infectious diseases, supportive in cancer care and in the elderly. In Kampo medicine (in contrast to most other forms of Eastern Medicines) philosophical considerations take a back seat to traditional clinical empiricism. This fact has stimulated the development of a lively dialogue and cooperation with Western forms of pharmacotherapy as well as clinical and basic research studies within the framework of “integrative medicine”, called “Tougouiryou” (統合医療). Due to this development as well as the ongoing international efforts to place Kampo into the context of Eastern Medicine in the WHO’s ICD-11 concept and the ISO TC-249 industrial standardisation process, Kampo is clearly the form of Eastern Medicine most easily accessible to Western thinking as this process has already happened in Japan during the adaptation of Western cultural techniques into Japanese civilisation in the Meiji reforms of the 19th century. It thus warrants a large-scale application in the West itself, where - especially in the context of our ageing society - new “integrative” forms of therapy for chronic diseases are urgently needed.

